# Effect of Glucocorticoid Administration in Intravenous Pulses on Selected Parameters of the Coagulation System

**DOI:** 10.1155/2022/3144685

**Published:** 2022-01-31

**Authors:** Przemysław Kłosowski, Renata Świątkowska-Stodulska, Dominik Stodulski, Mariusz Kaszubowski, Bartosz Karaszewski, Krzysztof Sworczak

**Affiliations:** ^1^Department of Endocrinology and Internal Medicine, Faculty of Medicine, Medical University of Gdańsk, Mariana Smoluchowskiego 17, Gdańsk 80-214, Poland; ^2^Department of Otolaryngology, Faculty of Medicine, Medical University of Gdańsk, ul. Mariana Smoluchowskiego 17, Gdańsk 80-214, Poland; ^3^Department of Statistics, Chair of Economic Sciences, Gdańsk University of Technology, ul. Gabriela Narutowicza 11/12, Gdańsk 80-233, Poland; ^4^Department of Adult Neurology, Faculty of Medicine, Medical University of Gdańsk, ul. Mariana Smoluchowskiego 17, Gdańsk 80-214, Poland

## Abstract

**Background:**

Changes of the coagulation system are promoted by serious infectious or noninfectious diseases, surgical procedures, and exogenous substances, including drugs. This study aimed to assess the effect of methylprednisolone pulses on selected parameters of the coagulation system.

**Methods:**

The study group consisted of patients suffering from multiple sclerosis, thyroid orbitopathy, or sudden sensorineural hearing loss. 48 patients and 20 healthy volunteers were examined. The hemostatic parameters: activity of coagulation factors (VIII, IX, and XI), antithrombin activity, protein C and S activity, and concentration of soluble tissue factor were analyzed at baseline and after 3 g and 5 g of methylprednisolone administration.

**Results:**

A statistically significant increase was noted in the activity of all the studied plasma coagulation factors, plasma coagulation inhibitors (except protein S activity), and the concentration of soluble tissue factor after methylprednisolone administration.

**Conclusion:**

The glucocorticoids administered in the intravenous pulses of methylprednisolone shift the balance toward thromboembolic complications.

## 1. Introduction

Glucocorticoids (GCs) are one of the most commonly used classes of drugs worldwide. A study conducted in the UK estimated that about 1% of the population is systemically treated with GCs, and a further increase in the use of these drugs is expected shortly [1]. Like any drug, GCs can cause side effects, depending on their dose, duration, and route of administration, as well as chemical structure [2]. Potential side effects of GC therapy also include hemostasis changes. It is difficult to determine the effect of GCs on the coagulation system as the initial disease entity which is an indication for the inclusion of GC therapy often modifies the hemostatic system, especially while exacerbated. The limited studies conducted so far do not clearly specify whether exogenous GCs enhance coagulation. In particular, there is lack of studies investigating the effect of potent intravenous GCs on the coagulation system, where it would be possible to distinguish the effect of an exacerbation of the underlying disease from the marked enhancement of coagulation resulting from the administration of GCs alone. These circumstances of course cannot relieve the physician from the obligation to assess the patient's thromboembolic risk. The authors of one study indicate that orally administered prednisolone induces a prothrombotic state in a healthy patient, which further suggests that GCs may increase the thromboembolic risk in patients with inflammatory diseases [3]. In this context, knowledge of the possible influence of GCs on the hemostasis process is crucial.

The main aim of this study was to evaluate selected parameters of the coagulation system, including the activity of coagulation factors (VIII, IX, and XI), activity of plasma coagulation inhibitors (antithrombin (AT), protein C (PC), and protein S (PS)), and concentration of soluble tissue factor (sTF), in patients subjected to methylprednisolone (MTP) pulse therapy due to medical indications. Despite urgent indications for intravenous administration of GCs, the study group consisted of patients with low thrombotic risk and initial diseases with low inflammatory potential.

## 2. Materials and Methods

### 2.1. General Information

This observational, prospective study was conducted in the following departments of the hospital of the Medical University of Gdańsk (University Clinical Center, Gdańsk): Department of Endocrinology and Internal Medicine, Department of Adult Neurology, and Department of Otolaryngology. The study was approved by the Bioethics Committee of the Medical University of Gdańsk (consent no. NKBBN/138/2016) and was conducted in accordance with the Declaration of Helsinki. All patients have given their informed consent for participation in the research study.

### 2.2. Selection of the Examined Subjects

The study group included adult patients treated in the abovementioned units of the hospital, who were subjected to systemic steroid therapy based on their medical indications (i.e., thyroid orbitopathy, sudden sensorineural hearing loss, or relapsing-remitting form of multiple sclerosis). The following were excluded from the study: patients with a history of thrombosis or pulmonary embolism in the last 6 months; patients receiving anticoagulant therapy; patients who underwent thrombolytic treatment in the past; patients treated with GCs orally and/or intravenously and/or intraarticularly in the last 4 weeks; patients diagnosed with connective tissue disease, thrombophilia, plasma hemorrhagic diathesis, active and clinically significant infection, chronic kidney disease with glomerular filtration rate <30, nephrotic syndrome, significant and clinically apparent liver damage, or active malignant neoplasm; patients undergoing chemotherapy or radiotherapy; and women who had used hormonal contraception or hormone replacement therapy in the last 3 months or were pregnant. Each patient was assessed on the Padua scale; all of the subjects received score indicating low thromboembolic risk. Therefore, none of the patients participating in the study received low-molecular-weight heparin or other anticoagulation agents during hospitalization. The control group consisted of healthy, adult volunteers who did not meet the exclusion criteria.

### 2.3. GC Treatment Scheme

**Figure 1:**
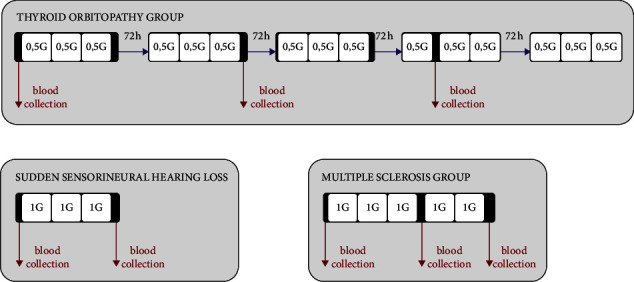
Scheme of methylprednisolone treatment in the study group based on the baseline disease entity.

All the patients in the study group were treated with intravenous MTP pulses. Patients who had a diagnosis of sudden hearing loss were given 1 g MTP per day for 3 consecutive days (cumulative dose 3 g), while those with relapsing multiple sclerosis were administered 1 g MTP per day for 5 consecutive days (cumulative dose 5 g). Patients diagnosed with thyroid orbitopathy received 0.5 g MTP per day for 3 consecutive days, followed by a break in therapy for the next 3 days, after which the MTP treatment was resumed (cumulative dose 7.5 g). Blood samples for the laboratory tests were taken at baseline as well after an administration of 3 g MTP (all groups) and 5 g MTP (those with thyroid orbitopathy and multiple sclerosis). Among patients with thyroid orbitopathy, three completed the examination before receiving 5 g MTP, for reasons beyond the investigator's control, and hence, the assessment was performed initially and after receiving 3 g MTP.  Figure 1 presents the flow diagram of the study.

### 2.4. Methodology

A standardized medical examination was conducted in each patient from the control and study groups, taking into account the age, gender, height and bodyweight of the patients, diagnosis of chronic diseases, and drugs used. The thromboembolic risk was assessed using the Padua scale [4]. Blood sample was collected from the brachial vein to determine the activity of factor: VIII, factor IX, factor XI, AT, PC, and PS; concentration of sTF; complete blood count and bilirubin (BIL) concentration; and activity of alanine aminotransferase (ALT) at baseline. After administration of a cumulative dose of 3 g of MTP (in all group) and 5 g of MTP (in thyroid orbitopathy and multiple sclerosis group), venous blood was collected again to determine the activity of factor VIII, factor IX, factor XI, AT, PC, and PS and the concentration of sTF. Blood samples after 3 and 5 g MTP administration were collected within 1-2 h after the last infusion. All the analyses were performed at the University Center for Laboratory Medicine in Gdańsk. The activity of coagulation factors, PS and APTT, was determined by the coagulometric method (with Siemens reagents, respectively, *Coagulation Factor VIII/IX/XI Deficient Plasma, Protein S Ac* and Pathromtin SL), while the activity of the remaining inhibitors of the coagulation system was determined by the chromogenic method (with Siemens reagents: *Berichrom Protein C*—for the determination of functionally active protein C; *Innovance Antithrombin* for the quantification of functionally active AT). The Siemens BCS analyzer was used for all of the analyses. The tissue factor (TF) concentration was determined after plasma thawing by ELISA using Cloud-Clone Corporation immunoenzymatic test (SEA 524 Hu, detection range: 15.6–1000 pg/ml, detection threshold <5.5 pg/ml). The complete blood count (Sysmex XN-10 automatic analyzer with dedicated Sysmex's reagents), ALT activity, and BIL concentration (determined by the spectrophotometric method on Abbot Architect C8000 analyzer with dedicated Abbot Alinity C alanine aminotransferase reagent kit and Alinity C total bilirubin reagent kit, respectively) were determined immediately after blood collection.

### 2.5. Statistical Analysis

The statistical analysis was conducted using STATISTICA 13.1 (Dell Inc. (2016), data analysis software system). All the data were meticulously analyzed and cleaned for outliers and possible measurement errors. In the case of small groups, the assumption of normality was checked using the strongest Shapiro–Wilk test or based on the graphical evaluation of a quantile chart. To examine the significance of differences in the mean values of unrelated variables, the parametric *t*-test (two-tailed) for independent samples with Welch correction was used to compare populations with different variances. For comparisons of mean values of dependent variables, the parametric paired *t*-test or Wilcoxon signed-rank test was conducted when appropriate. The relationship between qualitative data, including the occurrence fraction, was verified using an appropriate chi-square test. The significance level (*p*) was set at *α* = 0.05.

## 3. Results

A total of 48 patients were qualified for the study group, and 20 people were recruited to the control group.  Table 1 presents the demographic characteristics of the groups in total and by individual disease units. Collectively, the study group did not differ significantly from the control group in terms of age, BMI, and gender. The mean score on the Padua scale used for assessing thromboembolic risk did not differ significantly between the study and control groups (0.458 vs. 0.2, *P*=0.09), and all the observations (100%) were categorized as low thromboembolic risk.

**Figure 2:**
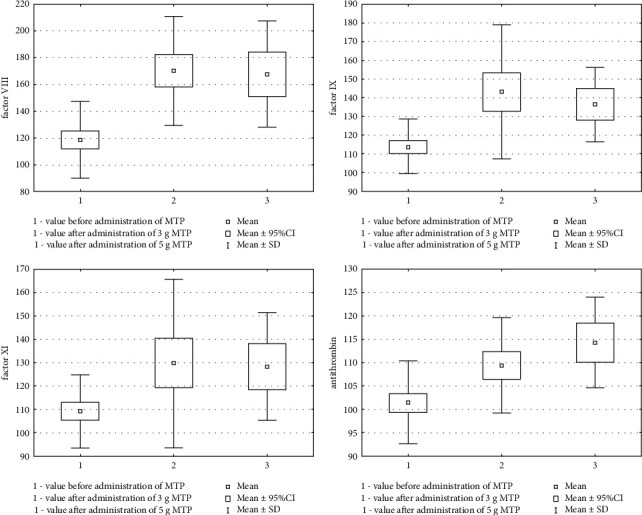
Comparison of changes in factors VIII, IX, and XI and antithrombin activity after the administration of 3 and 5 g methylprednisolone with the baseline values of the study group.

The baseline coagulation system parameters performed in the study and control group did not differ statistically significantly in terms of factor VIII, factor IX, factor XI, PC, PS activity, and sTF. Statistically significant differences were found in the activity of AT between the study and control groups, but the activity remained within the reference values in both groups.  Table 2 presents the comparison of coagulation system parameters at baseline (study and control groups). Changes in the coagulation system parameters after MTP administration were analyzed against the baseline values. After administration of 3 g MTP, a statistically significant change was found in the study group in the coagulation system parameters in relation to the initial parameters as follows: increase in the activity of factor VIII, factor IX, and factor XI; increase in the activity of AT and PC; and increase in sTF concentration. A comparative analysis of coagulation system parameters determined before MTP administration and after 5 g MTP administration was also performed. The directions of changes in the parameters were consistent with those observed after 3 g MTP administration. An increase in the activity of all the coagulation factors, AT, and PC and an increase in sTF concentration were observed. Tables 3 and 4 show a comparison of changes in the coagulation parameters after the administration of 3 g and 5 g of methylprednisolone, respectively, with the baseline values in the study group. In addition to the standard analysis of the mean values and medians, the percentage increase in the means and medians after administration of 3 and 5 g in relation to the baseline values was presented. The highest percentage increase was observed after the administration of 3 g MTP for factors VIII, IX, and sTF. Box plots were generated for significant statistical changes of selected coagulation system parameters (after 3 g and 5 g MTP administration) against baseline values (Figure 2). Due to the documented risk of thrombotic complications related to the activity of serum coagulation factors (VIII and IX) ≥150%, in Table 5, we present the percentage of patients in the study group who achieved such results after the administration of methylprednisolone.

Considering the differences in treatment regimens, the results were analyzed in the following subgroups: those with multiple sclerosis and sudden hearing loss together (the same treatment regimen) and those with thyroid orbitopathy. The analysis included baseline results and values recorded after 3 g MTP administration. It was demonstrated that the directions of changes in the coagulation parameters after 3 g MTP administration in both subgroups were identical to those observed during the analysis of the entire study population.

## 4. Discussion

This study showed that intravenous pulses of GCs increase the activity of plasma coagulation factors (VIII, IX, and XI), plasma coagulation inhibitors (AT and PC activity), and sTF concentration, which may shift the hemostatic balance toward thromboembolic complications.

While analyzing the impact of exogenous *hypercortisolemia*, it should be noted that patients requiring this therapy are usually burdened with diseases that may modify the coagulation system. Thus, it is extremely difficult to differentiate between thromboembolic complications, the etiology of which results from the basic disease process and those induced additionally, or perhaps first of all, by the GCs. Nevertheless, this study established broad exclusion criteria to control such extraneous variables. It is noteworthy that all patients in the study group were classified under low thromboembolic risk on the Padua scale. The strength of our study is also the fact that the baseline coagulation parameters in the study group did not differ significantly from the control group. This additionally indicates that the underlying disease entity did not significantly change the studied parameters of the coagulation system and allows to consider its influence on the obtained results as marginal.

Factor VIII, factor IX, and factor XI play significant roles among all the plasma coagulation cascade factors. The relationship between elevated concentrations of these coagulation factors and the increased incidence of venous thrombosis is well known [5–7]. Discussion of the obtained results in the context of the works of other researchers is difficult due to differences in the timing of blood collection, doses and type of steroid used, and the treatment regimen. Brotman et al. found an increase in factors VII, VIII, and XI in the group of healthy men who received 3 mg dexamethasone daily for 5 days compared to the group receiving placebo [8]. Their study showed the direct effect of GCs on the coagulation parameters. In a recent study, Miskiewicz et al. assessed the effect of intravenous pulse therapy with MTP on the coagulation system in patients with thyroid orbitopathy [9]. This study showed a reproducible and statistically significant increase in the activity of the plasma coagulation factor VIII 24 h and 48 h after the administration of 1, 6, and 12 pulses. In our study, after 3 and 5 g MTP administration, a clear and statistically significant increase in the activity of factor VIII, factor IX, and factor XI was observed against the baseline values. The observed changes in the range of factor VIII, factor IX, and factor XI were replicated in each of the studied subgroups and remained constant irrespective of the MTP treatment regimen used.

The risk of thrombosis is related to the level of factor VIII activity [10]. Koster et al. demonstrated that compared to the control group, the adjusted odds ratio for factor VIII >150% was 4.8 fold and comparable to the risk of factor V Leiden heterozygosity [11, 12]. In our research, initially elevated factor VIII activity >150% in the study group occurred in 14.58% of patients. After the administration of 3 g of MTP, the activity above 150% was observed in as much as 70.21%, while after the administration of 5 g, it was 58.33%. It has been also reported that high levels of coagulation factor IX are associated with an increased risk of venous thrombosis. Factor IX activity cutoffs that represent a higher thrombotic risk in research studies are different. In Lowe et al.'s study, a cutoff of 150% was used, and elevated factor IX activity was associated with a relative risk of venous thrombosis of 2.34 after adjustment for age, location, date of admission, and hormone replacement therapy use [13]. In our study, after the administration of 3 g of MTP, the increase in factor IX activity above the presented value was observed in 16.67% of patients in the study group and in 17.39% of patients after 5 g, while the initial activity above 150% was observed only in 4.17% of patients. In the presented study, the relative risk of a thromboembolic event was not assessed due to the lack of follow-up. High factor VIII levels may increase the risk of venous thrombosis via enhanced thrombin formation and/or induction of acquired APC resistance [10]. These observations are also related to studies on the influence of glucocorticoids on thrombin generation. Increased activation of the coagulation factors ultimately results in increased thrombin production. Majoor et al. showed that prednisone administered to healthy volunteers increased thrombin generation (measured as thrombin peak and rate index), which is a useful reflection of the procoagulant state [3].

The mechanism by which steroid hormones increase the concentration of plasma coagulation factors is yet to be determined. These actions are believed to take place by affecting gene transcription [14]. Assuming that the increase in the activity of coagulation factors depends exclusively on the genomic mechanism, a further increase in their activity should be expected along with an increase in the cumulative dose of the administered steroid. However, this was not observed in the present study. It is also difficult to explain such a rapid increase in plasma coagulation factors after the first pulse of MTP (blood collected 1-2 h after the end of MTP infusion) by the genome mechanism. These arguments may indicate the predominance of nongenomic mechanisms of GC activity in the activation of the coagulation system and should be considered while designing further scientific analyses.

The present study also evaluated the influence of GCs on the activity of natural coagulation system inhibitors. These include AT, protein C and S systems, and TF pathway inhibitor protein. AT belongs to the Serpin family and is encoded by the SERPINC1 gene. Its main role is to inactivate the activated factor Xa and thrombin as well as activated factors IXa, XIa, XIIa, and VIIa associated with TF [15, 16]. When thrombin appears in the bloodstream, the protein C system consisting of protein C, its cofactor S, thrombomodulin, and the endothelial protein C receptor are also activated. The final activation of protein C causes the inhibition of blood coagulation through partial proteolysis of activated factors Va and VIIIa [15]. In this study, a significant increase was found in the activity of AT and PC after 3 and 5 g MTP administration. Concerning changes in the natural coagulation system inhibitors, two questions arise: which mechanism of GC activity causes this system activation? And also, what is the pathophysiological significance of the observed changes? The available literature has not yet assessed the activity of AT, PC, and PS when the initial indication for systemic administration of GCs was diseases with low inflammatory potential. There is also a lack of studies assessing the activity of the natural coagulation inhibitors after the administration of GCs in healthy volunteers. Most researchers have assessed the effect of GCs on the analyzed parameters in patients burdened with diseases of high inflammatory potential or modifying the coagulation system *per se.* In patients with diagnosed systemic lupus erythematosus, the activity of PC, PS, and AT was found to be increased after prednisolone administration [17]. Oner et al. demonstrated a statistically significant increase in APC and APS in children with idiopathic thrombocytopenic purpura who received MTP in high doses (30 mg/kg/24 h orally, one dose for 7 days) [18]. Barettino et al. demonstrated that GCs can modulate AT expression by acting as nuclear hormone receptor agonists [19]. More importantly, identical directions of changes in APC, APS, and AT were observed in patients with Cushing's syndrome, in whom the risk of thromboembolism is significantly higher than in the general population [20]. It is suggested that the observed changes in APC, APS, and AT may be secondary to the increased activity of the coagulation factors—mainly factor V and factor VIII—which may be a compensation mechanism for counteracting the increased plasma coagulation activity [20, 21].

This study also established the influence of GCs on the process of coagulation system initiation expressed by an increase in sTF concentration. TF is the main initiator of the coagulation cascade. As a cofactor, it significantly increases the catalytic activity of factor VIIa [22]. Apart from our research, a relatively small number of studies assessing the effect of GCs on TF concentration are available. It was proved that TF circulating in the blood—the so-called “blood-borne TF”—can be active and exert procoagulation effects [23]. After both 3 and 5 g MTP administration, a significant increase in sTF concentration was observed against the baseline values. The sTF results were not affected by the GC treatment regimen, and higher sTF values were observed against the baseline values after the administration of 3 g GCs in patients treated with short and extended regimens. The main direction of sTF concentration change seems to be consistent with the observations of other researchers. De Kruif et al. studied changes occurring in the coagulation parameters after the administration of lipopolysaccharide (LPS) *Escherichia coli* in patients who had previously received prednisolone [24]. Moreover, the increase in sTF in response to LPS was higher in the group which received GCs.

## 5. Conclusions

This study is one of the few studies in which, despite the urgent indications for corticotherapy, a study group was selected with initially low inflammatory potential as well as low thrombotic risk. Based on the statistical analysis of changes in the coagulation parameters after 3 and 5 g MTP administration and comparison of the results with the control group, the following conclusions are made:

1. The coagulation system in patients suffering from multiple sclerosis, thyroid orbitopathy, or sudden sensorineural hearing loss treated with MTP pulse undergoes significant changes after 3 and 5 g administrations of the drug.
2. The initiation of the blood coagulation process expressed in sTF concentration is intensified after MTP administration.
3. The administration of GCs in intravenous pulses increases the activity of plasma coagulation factors—factor VIII, factor IX, and factor XI.
4. During intravenous pulses of GCs therapy, an increase in the activity of plasma coagulation inhibitors is observed.

## Figures and Tables

**Table 1:** Demographic characteristics of the study and control groups.

Variable	Study group	Multiple sclerosis	Thyroid orbitopathy	Sudden hearing loss	Control group
Number of patients	48	10	17	21	20
Women/men	29/19	8/2	15/2	6/15	10/10
Median age (Q_1_–Q_3_)	57.00 (17–77)	44.00 (23–63)	60.00 (33–77)	55.00 (19–68)	44.00 (24–67)
Mean BMI (SD)	27.25 (4.48)	25.71 (4.86)	28.02 (4.91)	27.36 (4.21)	25.49 (3.60)
Mean Padua scale (SD)	0.46 (0.82)	0.80 (1.23)	0.41 (0.62)	0.33 (0.73)	0.23 (0.43)

BMI = body mass index (kg/m^2^).

**Table 2:** Comparison of coagulation system parameters of the study and control groups before methylprednisolone administration.

Variable	Control (*n* = 20)^∗^	Study (*n* = 48)
	Mean (SD)	Mean (SD)	*P*
Factor VIII	118.40 (32.35)	118.73 (26.94)	0.968
Factor IX	110.50 (14.14)	115.73 (14.67)	0.178
Factor XI	108.35 (12.08)	109.56 (16.94)	0.740
AT	96.55 (10.61)	103.44 (7.19)	0.013
PC act.	117.40 (20.77)	119.42 (17.95)	0.707
PS act.	81.30 (14.92)	82.42 (21.65)	0.808
sTF	139.32 (42.87)	130.06 (39.15)	0.421
APTT	29.85 (3.66)	29.13 (3.09)	0.443

^∗^For sTF *n* = 19, due to the extremely high sTF values, significantly different from the rest of the measurements, in one patient of the study group, the result was not taken into account in the statistical analysis. APTT = activated partial thromboplastin time; AT = antithrombin; PC act. = protein C activity; PS act. = protein S activity; sTF = soluble tissue factor.

**Table 3:** Comparison of changes in coagulation system parameters after 3 g methylprednisolone administration with the baseline values in the study group (*N* = 48)^∗^.

Variable	Mean/median	Baseline	After 3 g		
		Measures	Measures	%^∗∗^	*P* ^∗∗∗^
Factor VIII	Mean (SD)	118.73 (26.94)	170.06 (40.97)	+43.23%	<0.001
	Median (Q_1_–Q_3_)	115.50 (98.50–132.00)	166.00 (147.00–197.00)	+43.72%	<0.001

Factor IX	Mean (SD)	115.73 (14.67)	143.35 (35.85)	+23.87%	<0.001
	Median (Q_1_–Q_3_)	114.50 (106.00–122.50)	139.50 (128.00–147.00)	+21.83%	<0.001

Factor XI	Mean (SD)	109.56 (16.94)	129.73 (35.98)	+18.41%	<0.001
	Median (Q_1_–Q_3_)	107.50 (99.00–120.50)	122.50 (105.50–143.50)	+13.95%	<0.001

AT	Mean (SD)	103.44 (7.19)	109.44 (10.25)	+5.80%	<0.001
	Median (Q_1_–Q_3_)	104.00 (98.00–108.50)	109.50 (102.00–118.50)	+5.29%	<0.001

PC act.	Mean (SD)	119.42 (17.95)	146.33 (29.49)	+22.53%	<0.001
	Median (Q_1_–Q_3_)	119.50 (105.50–133.00)	139.00 (128.00–160.00)	+16.32%	<0.001

PS act.	Mean (SD)	82.42 (21.65)	85.48 (22.45)	+3.71%	0.335
	Median (Q_1_–Q_3_)	83.50 (68.00–96.50)	85.50 (73.00–96.00)	+2.40%	0.076

sTF	Mean (SD)	130.06 (39.15)	177.50 (70.71)	+36.48%	<0.001
	Median (Q_1_–Q_3_)	135.00 (97.50–159.50)	175.00 (124.00–215.00)	+29.63%	<0.001

APTT	Mean (SD)	29.13 (3.09)	25.69 (2.43)	−11.81%	<0.001
	Median (Q_1_–Q_3_)	28.00 (27.00–30.50)	25.00 (24.00–27.00)	−10.71%	<0.001

^∗^For factor VII after 3 g *n* = 47, details in the text, ^∗∗^ percentage change of mean or median over baseline, ^∗∗∗^*P* value appropriately for paired *t*-test or Wilcoxon signed-rank test. APTT = activated partial thromboplastin time; AT = antithrombin; PC act. = protein C activity; PS act. = protein S activity; sTF = soluble tissue factor.

**Table 4:** Comparison of changes in coagulation system parameters after 5 g methylprednisolone administration with the baseline values in the study group (*N* = 24)^∗^.

Variable	Mean/median	Baseline	After 5 g		
		Measures	Measures	%^∗∗^	*P* ^∗∗∗^
Factor VIII	Mean (SD)	112.67 (25.74)	167.58 (39.64)	+48.74%	<0.001
	Median (Q_1_–Q_3_)	106.50 (96.50–128.00)	162.00 (134.50–193.00)	+52.11%	<0.001

Factor IX	Mean (SD)	116.87 (17.04)	136.70 (19.92)	+16.97%	<0.001
	Median (Q_1_–Q_3_)	117.00 (108.50–124.00)	140.00 (124.00–147.00)	+19.66%	<0.001

Factor XI	Mean (SD)	108.58 (17.66)	128.42 (23.13)	+18.27%	<0.001
	Median (Q_1_–Q_3_)	105.50 (97.50–119.00)	125.00 (114.50–135.50)	+18.48%	<0.001

AT	Mean (SD)	103.71 (7.98)	114.25 (9.74)	+10.16%	<0.001
	Median (Q_1_–Q_3_)	107.00 (97.50–109.00)	115.50 (108.00–123.00)	+7.94%	<0.001

PC act.	Mean (SD)	116.50 (15.49)	149.88 (27.38)	+28.65%	<0.001
	Median (Q_1_–Q_3_)	118.00 (101.50–129.50)	147.50 (127.50–176.00)	+25.00%	<0.001

PS act.	Mean (SD)	80.54 (21.07)	82.42 (17.59)	+2.33%	0.559
	Median (Q_1_–Q_3_)	84.50 (65.50–94.00)	83.50 (72.50–93.50)	−1.18%	0.513

sTF	Mean (SD)	128.13 (42.19)	152.42 (70.43)	+18.96%	0.029
	Median (Q_1_–Q_3_)	116.00 (95.00–167.50)	154.00 (101.00–205.00)	+32.76%	0.029

*APTT*	Mean (SD)	29.54 (3.19)	25.17 (2.43)	−14.79%	<0.001
	Median (Q_1_–Q_3_)	28.00 (28.00–30.50)	25.00 (24.00–26.00)	−10.71%	<0.001

^∗^For factor IX after 5 g *n* = 23, details in the text, ^∗∗^percentage change of mean or median over baseline, ^∗∗∗^*P* value appropriately for paired *t*-test or Wilcoxon signed-rank test. APTT = activated partial thromboplastin time; AT = antithrombin; PC act. = protein C activity; PS act. = protein S activity; sTF = soluble tissue factor.

**Table 5:** Coagulation factors VIII and IX activity (results ≥150%).

Variable	Baseline	After 3 g	After 5 g
	Number of patients (%)	Number of patients (%)	Number of patients (%)
Factor VIII	7 (14.58%)	33 (70.21%)	14 (58.33%)
Factor IX	2 (4.17%)	8 (16.67%)	4 (17.39%)

## Data Availability

Basic data confirming the results of our study are stored at the Endocrinology Department, Medical University of Gdańsk, Poland, and are available by e-mail upon request.

## Abbreviations

ALT: Alanine aminotransferase
APTT: Activated partial thromboplastin time
AT: Antithrombin
BIL: Bilirubin
BMI: Body mass index
GCs: Glucocorticoids
LPS: Lipopolysaccharide
MTP: Methylprednisolone
PC: Protein C
PS: Protein S
sTF: Soluble tissue factor
TF: Tissue factor.
